# Neuromyelitis Optica in Pregnancy Complicated by Posterior Reversible Encephalopathy Syndrome, Eclampsia and Fetal Death

**DOI:** 10.14740/jocmr2031w

**Published:** 2014-12-29

**Authors:** Catherine Igel, Diana Garretto, Matthew S Robbins, Michael Swerdlow, Nancy Judge, Ashlesha Dayal

**Affiliations:** a Department of Obstetrics and Gynecology and Women’s Health, Albert Einstein College of Medicine/Montefiore Medical Center, Bronx, NY, USA; b Department of Neurology, Albert Einstein College of Medicine/Montefiore Medical Center, Bronx, NY, USA

**Keywords:** Aquaporin 4, Devic’s disease, Eclampsia, Posterior reversible encephalopathy syndrome, Neuromyelitis optica

## Abstract

Neuromyelitis optica (NMO) is a demyelinating syndrome characterized by optic neuritis and acute myelitis with poor recovery and a progressive course. We report a poor outcome complicated by posterior reversible encephalopathy syndrome (PRES) and eclampsia and review available literature and current evidence for anticipation of adverse fetal and maternal effects. After a pregnancy complicated by multiple admissions for painful NMO exacerbations, a primiparous patient with seropositive NMO presented at 31 + 3/7 weeks with eclampsia, HELLP and subsequent fetal death. MRI confirmed PRES. NMO may be associated with eclampsia and leads to adverse maternal and fetal outcomes. Posited mechanisms include antibody-mediated placental damage and a heightened risk of eclampsia-associated PRES. Further characterization of the course of NMO and its relationship with pregnancy outcomes in larger series would be invaluable.

## Introduction

Neuromyelitis optica (NMO), also known as Devic’s disease, is an autoimmune syndrome largely characterized by optic neuritis and myelitis with poor recovery and a progressive course [1]. NMO had previously been considered a multiple sclerosis (MS) subtype until Wingerchuk et al identified a biomarker not found in MS, NMO-IgG targeting a membrane-bound water channel transporter protein, aquaporin-4 [2]. Recent evidence supports a humoral pathogenetic mechanism [3]; criteria for NMO now include NMO-IgG positivity, optic neuritis, a longitudinal spinal cord lesion of at least three segments and an initial cerebral MRI non-diagnostic for MS [3]. NMO is much less common than MS but similarly affects women of childbearing age and presents diagnostic and management challenges in pregnancy due to its association with early losses and postpartum exacerbations. In spite of an uneventful pregnancy preceding diagnosis and stable disease at conception, we present a patient with well-established preconception NMO who experienced refractory symptoms, eclampsia and fetal loss.

## Case Report

**Figure 1:**
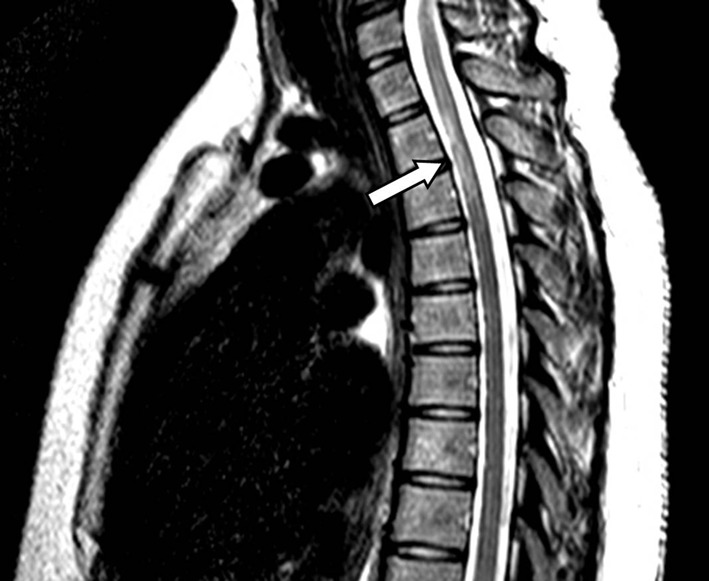
Sagittal T2-weighted MRI of the spine reveals a hyperintense lesion (arrow) at the T1-T3 levels of the spinal cord.

**Figure 2:**
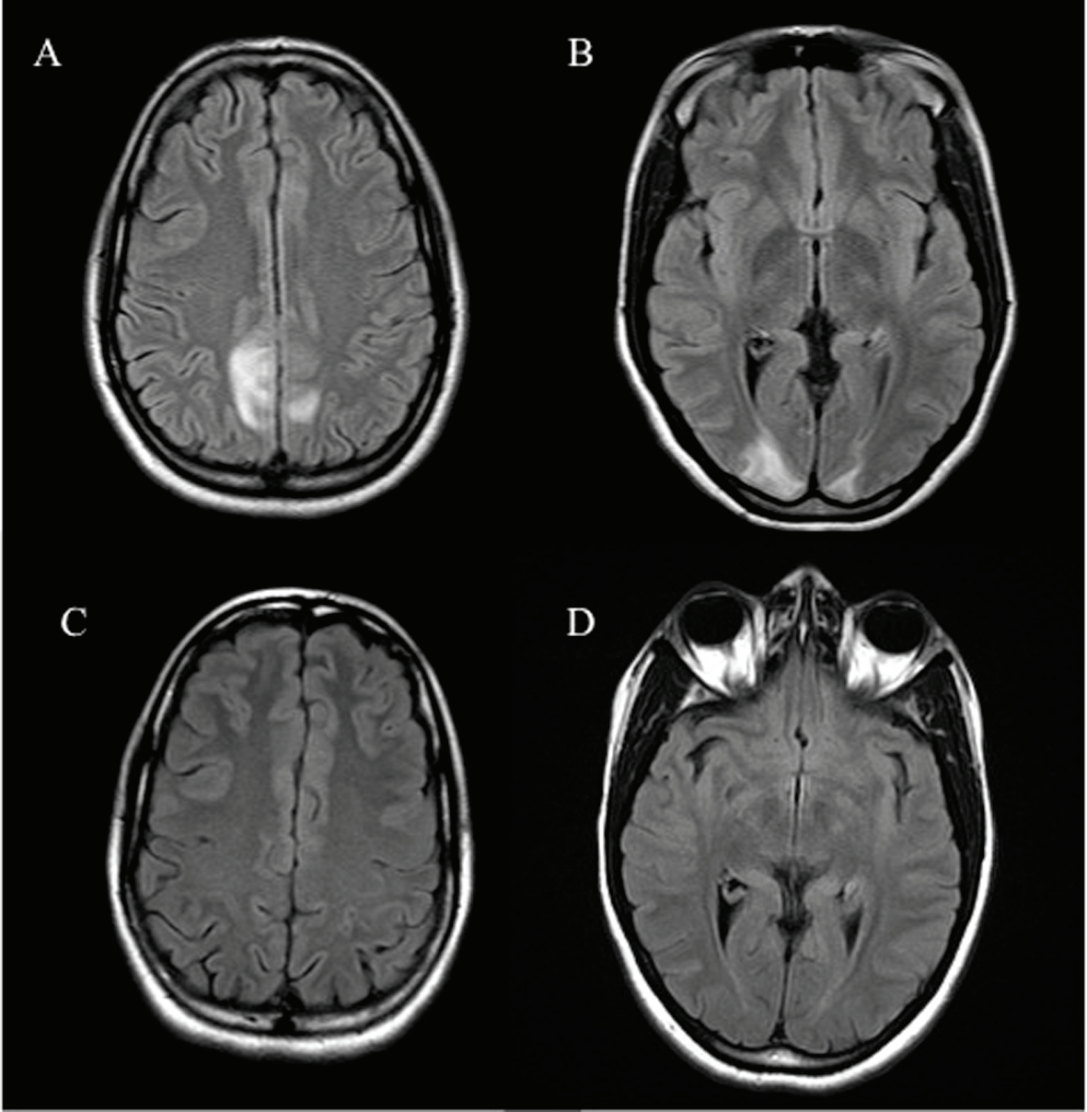
Axial FLAIR sequence MRI of the brain reveals hyperintensities in the parieto-occipital regions (A, B) which resolved on repeat MRI (C, D).

A 27-year-old African American G4P1021 presented to the labor evaluation suite at 31 + 3/7 weeks, complaining of headache, nausea, vomiting, visual flashes and unstable gait. NMO was diagnosed 3 years prior to the current pregnancy (Fig. 1), characterized by transient unilateral blindness, and a T1-T3 demyelinating spinal cord lesion, initially identified as MS, but with normal CNS MRI and CSF profile. Malar rash and positive ANA raised suspicion for co-existing SLE, but other characteristic findings were absent. NMO was diagnosed after positive anti-NMO IgG serologic testing. Symptoms of neuralgic back pain, impaired vision and ambulation resolved preconception on prednisone, azathioprine and gabapentin. The patient independently discontinued all medications in the first trimester, with relapses starting at 17 weeks. Plasmapheresis, initially successful, caused severe hypofibrinogenemia and was discontinued in favor of prednisone and gabapentin, with azathioprine and carbamazepine added during later admissions. The patient also had bipolar disease, stable off lithium during gestation. Obstetric history included two elective abortions and an uncomplicated term vaginal birth 6 years earlier. Once symptoms were controlled, pregnancy proceeded uneventfully with normal fetal testing. She was initially alert and oriented, with identified fetal heart tones, BP 135/87; within minutes of arrival, she experienced three tonic/clonic seizures with hypertension of 176/100. Fetal death occurred during initial maternal stabilization using intravenous magnesium sulfate and labetalol. Laboratory revealed elevated hepatic enzymes (twice baseline) and depressed platelet count, nadir of 111,000/μL. Cranial CT demonstrated bilateral paramedian parietal lobe hypodensities suspicious for posterior reversible encephalopathy syndrome (PRES) (Fig. 2). Induction with misoprostil achieved vaginal delivery of a non-anomalous stillborn 1,140 g female. Placental pathology showed acute inflammation of fetal membranes, numerous intervillous thrombi and focal Tenney-Parker change (vascular knotting) consistent with maternal hypoperfusion. Brain MRI confirmed hyperintensities in the bilateral parietal and occipital regions consistent with PRES. The patient’s sensorium cleared; she remained in remission from NMO through 3 months postpartum. Brain MRI repeated several months later confirmed resolution of the lesions.

## Discussion

The cited incidence of NMO is between 1 and 4.4 cases per 100,000, with a female/male ratio of 9:1 and commonly features a relapsing pattern [4]. The prevalence is quite difficult to ascertain due to extreme heterogeneity between studies. However, the prevalence was lowest in Cuba at 0.52 and the highest in South Denmark at 4.4 per 100,000 [5]. A Brazilian group posited that African heritage predisposes to more aggressive disease [4]. NMO is associated with connective tissue disorders; workup should include antinuclear antibody, anti-ds DNA, lupus anticoagulant, antiphospholipid antibodies, NMO-IgG, CNS and spine MRI and cerebrospinal fluid analysis. Therapeutic goals include induction of remission, disease stabilization and symptom minimization; steroids, a variety of other immunosuppressive drugs and plasmapheresis have been beneficial. Reasonable preconception counseling to avoid known teratogens and to continue effective therapies is not evidence-based.

NMO in pregnancy has been poorly characterized. As NMO is known to be a TH2-mediated disease, the concern is that cytokines secreted in pregnancy may change NMO activity. TH2 cytokines are synthesized in pregnancy by the fetoplacental unit. Interestingly, Bourre et al noted in their retrospective study a decrease in the relapse rate during the first and second trimester, but worsening in the third trimester and into the postpartum period up to a year. Therefore, the pathophysiology is unlikely to be only due to the increase in cytokines [6]. The biomarker for NMO, the antibody to aquaporin-4, is found in placenta as well as neural tissue, with NMO-IgG binding sites peaking at 21 weeks. In a mouse model, NMO-IgG with human complement activation and deposition of membrane attack complexes resulted in placental inflammation and fetal wastage [7]. A relationship between placental damage, NMO-IgG and disease activity has not yet been identified in humans.

The outcomes of pregnancies have also been difficult to characterize as still very few reports of NMO in this time period are available. Fragoso et al found that six out of 17 patients had an uneventful pregnancy, five of 17 had relapses in the pregnancy, and some had a variety of poor outcomes in individual patients including hemorrhagic stroke, preeclampsia, a miscarriage, and vaginal bleeding in the pregnancy. Fetal outcomes were only collected on eight of the 17 patients but with no abnormalities noted.

Although discontinuing treatment likely triggered disease relapse in our patient, the etiology of eclampsia and PRES is less obvious. Interestingly, PRES is seen in NMO spectrum disorders without the provocation of eclampsia, but as PRES is commonly seen in patients who suffered from eclampsia, there may be additive risk factors in pregnancy. Magana et al postulate that the aquaporin-4 antibody is implicated in the pathogenesis of PRES due to its control in the CNS of bidirectional water flux in the brain [7].

Further characterization of the course of NMO and its relationship with pregnancy outcomes in larger series would be invaluable. Until then, pregnant women with NMO should be advised about the risks of withholding immunosuppression and the potential added risk of preeclampsia and eclampsia. Vigilant antepartum monitoring seems appropriate in women with NMO, particularly with active disease activity, including education about the early signs and symptoms of preeclampsia and eclampsia.
